# iSVP: an integrated structural variant calling pipeline from high-throughput sequencing data

**DOI:** 10.1186/1752-0509-7-S6-S8

**Published:** 2013-12-13

**Authors:** Takahiro Mimori, Naoki Nariai, Kaname Kojima, Mamoru Takahashi, Akira Ono, Yukuto Sato, Yumi Yamaguchi-Kabata, Masao Nagasaki

**Affiliations:** 1 Department of Integrative Genomics, Tohoku Medical Megabank Organization, Tohoku University, 2-1 Seiryo-machi, Aoba-ku, Sendai, Miyagi, 980-8573, Japan

## Abstract

**Background:**

Structural variations (SVs), such as insertions, deletions, inversions, and duplications, are a common feature in human genomes, and a number of studies have reported that such SVs are associated with human diseases. Although the progress of next generation sequencing (NGS) technologies has led to the discovery of a large number of SVs, accurate and genome-wide detection of SVs remains challenging. Thus far, various calling algorithms based on NGS data have been proposed. However, their strategies are diverse and there is no tool able to detect a full range of SVs accurately.

**Results:**

We focused on evaluating the performance of existing deletion calling algorithms for various spanning ranges from low- to high-coverage simulation data. The simulation data was generated from a whole genome sequence with artificial SVs constructed based on the distribution of variants obtained from the 1000 Genomes Project. From the simulation analysis, deletion calls of various deletion sizes were obtained with each caller, and it was found that the performance was quite different according to the type of algorithms and targeting deletion size. Based on these results, we propose an integrated structural variant calling pipeline (iSVP) that combines existing methods with a newly devised filtering and merging processes. It achieved highly accurate deletion calling with >90% precision and >90% recall on the 30× read data for a broad range of size. We applied iSVP to the whole-genome sequence data of a CEU HapMap sample, and detected a large number of deletions, including notable peaks around 300 bp and 6,000 bp, which corresponded to Alus and long interspersed nuclear elements, respectively. In addition, many of the predicted deletions were highly consistent with experimentally validated ones by other studies.

**Conclusions:**

We present iSVP, a new deletion calling pipeline to obtain a genome-wide landscape of deletions in a highly accurate manner. From simulation and real data analysis, we show that iSVP is broadly applicable to human whole-genome sequencing data, which will elucidate relationships between SVs across genomes and associated diseases or biological functions.

## Background

Structural variation (SV) is one of the key features of genetic variations among individuals. SV includes several types of sequence-level polymorphisms such as insertions, deletions, inversions, translocations, and duplications or copy number variations. A number of studies have implicated relationships between such SVs and human phenotypes including diseases such as cancer susceptibility [1], mental disorders [2], metabolic disorders [3], and some types of intractable diseases [4-6].

While most single nucleotide polymorphisms (SNPs) are di-allelic and easier to detect, many SVs are multi-allelic in general and their patterns vary significantly among different SV types [7]. Consequently, the detection of SVs is much more difficult than that of SNPs. Large-scale genomic SVs have conventionally been investigated by Southern blot analysis. In later years, fluorescence *in situ *hybridization to DNA fibers (Fiber-FISH), which is based on the hybridization of fluorescent probes onto chromosomes, has been widely used for the detection of SVs [8,9]. Such large genomic deletions in specific chromosomal regions have been reported to be associated with severe neuropathy and neurocognitive deficits [10,11]. The development of microarray technologies, such as array comparative genomic hybridization (array-CGH) and whole-genome SNP genotyping technologies, has enhanced the study of human SVs at the genome-wide level by detecting gains and losses of DNA regions compared to the reference genome [12-14]. A high-resoultion statistical method to detect SVs with a hidden Markov model from Illumina high-density SNP genotyping data has been proposed [15]. However, there are limitations to array-based methods for SV detection. First, because the SNP probes of these arrays do not uniformly represent SVs distributed across the whole genome, some SVs outside the targeted region might not be detected at all. Second, the arrays can only detect SVs of relatively large sizes covering more than several kilobases. Third, they cannot detect the precise breakpoints of the SVs. Finally, novel insertions cannot be detected since they are not pre-included in array probes.

Recent progress in NGS technologies have enabled us to detect SVs more directly. More recently, several types of computational methods based on NGS data have been proposed for finding SVs with higher resolution than SNP array-based methods. In these analyses, typically 35-100 bp paired-end reads are mapped to the reference genome, and SVs are inferred from the status of the mapped reads. The first approach, called read depth (RD), utilizes the depth of coverage of mapped reads [16]. Essentially, lower and higher depth values imply deletions and duplications of the region, respectively. The second approach, read pair (RP), uses anomalous paired-end mappings of reads [17]. According to the separation distance and read orientation, SVs can be inferred. The third strategy, split read (SR), evaluates partial mapping of reads; this is employed by Pindel [18] and ClipCrop [19]. Pindel uses a portion of paired end reads in which one of the pair is unmapped. On the other hand, ClipCrop uses 'soft-clipped' reads, in which a part of the read maps to the reference genome and the other does not. The soft-clip information can be obtained from the mapped result encoded in the Sequence Alignment/Map (SAM) format [20]. The fourth approach, sequence assembly (AS), assembles novel sequences from short reads locally. However, currently, there appear to be only a few integrative tools to detect all kinds of SVs for different types and size, and the characteristics and performance of these various tools have not yet been extensively studied. Recently, whole-genome sequencing data of many individuals have been produced very rapidly, as in the 1000 Genomes Project [21]. Hence, the development of a reliable and robust SV detection method from whole-genome sequencing data is urgently needed.

First, we evaluated the performance of several SV detection tools with simulated paired-end sequencing data for various deletion sizes. Based on the evaluation, it would be possible to gain >90% precision and recall for a broad range of deletion sizes by combining different types of algorithms in a straightforward way. However, deletions detected by different callers often contain multiple entries for the same deletions, and these entries may have differences in their sizes, positions, and their reliablities. Thus, a naïve combination of multiple callers' results may fail to produce accurate detection calls. We propose the **i**ntegrated **S**tructural **V**ariant calling **P**ipeline (iSVP), which combines existing SV detection methods and resolves this problem by selecting a reliable subset of deletion calls and unifying duplicated entries. A tool based on a similar concept has been proposed, named SVMerge [22]. The tool also combines SV detected results from multiple callers and generates non-redundant calls like iSVP. The tool handles SVs other than deletions, but the size of the results is restricted to >100 bp. iSVP handles smaller deletions consistently and our procedure in the merging step does not depend on deletion size (see Methods section). In addition, the parameters employed in filtering and merging steps of iSVP are determined by evaluating simulation data for a wide range of sizes.

We also investigate the relationship between depth of coverage and SV detection performance with our pipeline, and show that high coverage sequencing (more than 20×) is necessary to obtain good performance in SV detection in the simulation experiment. Finally, we apply our proposed pipeline to whole genome sequencing data obtained from an NA12878 sample with an average depth of 45× and present a comprehensive picture of deletion events in which the resolution ranges from 1 bp to more than 100,000 bp. We also confirm that some of the predicted deletions with our pipeline have been validated in several independent experiments [23-25] and that its performance is equivalent to or better than that of tools used independently for all the datasets.

## Methods

### Evaluation of SV detection algorithms

We first compare and evaluate the performance of existing deletion callers from the synthetically generated NGS read data with various ranges of deletion size and depth of coverage. Typically, SV detection algorithms are classified into the following types: read depth (RD), read pair (RP), split read (SR), assembly (AS), and combinations of those algorithms. Table 1 summarizes the characteristics of deletion callers used in our comparison. BreakDancer (BD) [17] is classified as an RP-type tool that uses discordant read pairs (a pair of reads that are not properly aligned) to detect SVs. This method uses a distribution of fragment lengths of paired-end reads to find anomalous read pairs. Its computational cost is much lower than that in other algorithms, and hence, easily applicable to find large deletion sizes. Pindel [18] is an SR-type tool that uses part of paired end reads in which one of the pair is unmapped. It splits each unmapped read and determines the break points of SVs by an algorithm called pattern growth approach. Delly [26] uses a combination of RP, RD, and SR approaches. GATK Haplotype Caller (HC) [27,28] is an AS-type method that performs local *de novo *assembly of haplotypes via de Bruijn graphs to detect SNPs and indels at base-pair resolution. However, the method needs a large amount of computational resources in terms of both memory space and CPU time. In our computational analysis, we used BD Max version 1.1, Delly version 0.0.9, Pindel version 0.2.4, and HC of GATK version 2.5-2.

**Table 1:** Summary of SV detection tools.

Tool	Algorithm	Detectable SV types	Simulation 30× CPU time, max. memory size	NA12878 45× CPU time, max. memory size
BD	RP	DEL, INS, INV, TRA	1.6h, 0.3Gb	2.1h, 0.5Gb
Delly	RP, RD, SR	DEL, INV, DUP, TRA	1.3h, 0.5Gb	24h, 9Gb
Pindel	SR	DEL, INS, INV, DUP, TRA	19h, 3Gb	37h, 3Gb
HC	AS	DEL, INS, other	68h, *9Gb	180h, *9Gb

SV detection tools are summarized according to algorithm types, detectable SV types, and computational resources required in our analyses. For each tool, CPU time and maximum memory size were measured for the 30× simulation data and the 45× whole genome sequence data of NA12878. RP, RD, SR, and RP stand for read pair, read depth, split read, and assembly approaches, respectively. 'BD' is BreakDancer. HC, Haplotype Caller; 'DEL', deletion; 'INS', insertion; 'INV', inversion; 'DUP', duplication; 'TRA', translocation.

* HC was performed with explicitly specified maximum memory size.

### Simulation data preparation

**Figure 1:**
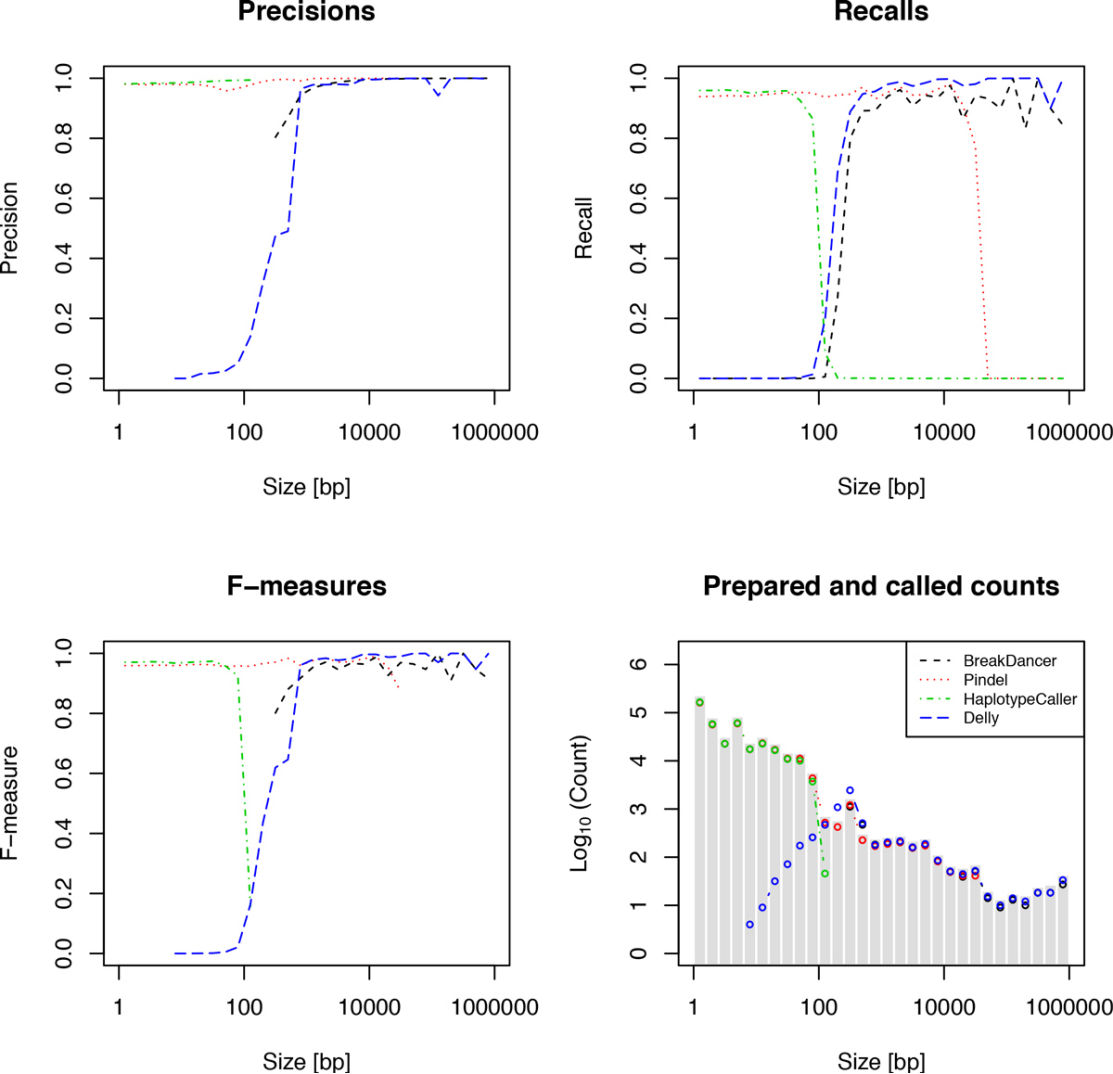
**Deletion calling performance**. The left-top, right-top, left-bottom, and right-bottom panels show precisions, recalls, F-measures, and the numbers of deletion calls, respectively, using simulation data with an average read depth of 30×. The right-bottom panel also shows the histogram of prepared deletions for each size.

We prepared an artificial human genome sequence by adding SVs, insertions, and deletions to the reference genome hg19 at randomly selected regions. The size of the SVs follows the size distribution shown in the histogram at the bottom-right corner of Figure 1, which was constructed based on SV calling results from sequencing data in the 1000 Genomes Project [21,29]. We then synthetically generated 100-bp paired-end reads from the genome sequence and prepared a set of simulated sequence data with average depths of 5×, 10×, 20×, and 30×. The insert size of paired-end reads was set to follow a normal distribution with a mean of 350 and a standard deviation of 50, and a 0.1% substitution error was considered at each nucleotide position. For paired-end mapping of the simulated data, we used Burrows-Wheeler Aligner (BWA) [20] with the default options. The resultant SAM file was then used in subsequent SV callers.

### Evaluation metrics for deletion calls

**Figure 2:**
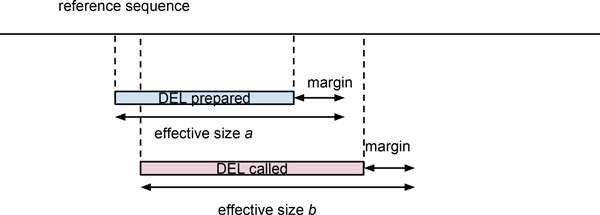
**An evaluation metric for deletion calls**. For the evaluation of SV calling performance, a quality score was defined for each deletion call for overlaps of called regions. See Methods section for details.

We defined the precision and recall of deletion calls for given size *s *as follows:

$$\text{precision}\;\text{(}s\text{)}=\underset{i\in \left\{\text{all called SVs with size}\;=\;s\right\}}{\sum }\underset{\text{j}\in \text{\{ all prepared SVs\}}}{\text{max}}q_{ji}/N\left(\text{called}\;\text{size}\;\text{ = }\;s\right),$$

$$\text{recall}\;\text{(}s\text{)}=\underset{i\in \left\{\text{all prepared SVs with size}\;=\;s\right\}}{\sum }\underset{\text{j}\in \text{\{ all called SVs\}}}{\text{max}}q_{ij}/N\left(\text{prepared}\;\text{size}\;\text{ = }\;s\right),$$

where *N *is the number of called or prepared SVs and *q *is a quality value that is defined for each overlap between prepared SVs and called SVs, and takes a value between 0 and 1. The quality *q *is defined as:

$$q_{ij}=\text{size}\;\text{(}a_{i}\cap b_{j}\text{)/size}\;\text{(}a_{i}\cup b_{j}\text{)},$$

where *a *and *b *are the effective regions of called and prepared SVs, respectively. The effective region is extended from the actual region by a fixed length margin as shown in Figure 2. The margin is introduced in order to retain SV calls that were correct but slightly deviated from the actual SV region due to ambiguity of mapping to the reference genome, often observed at interspersed repeats and low-complexity regions. We used 50 bp for the margin in our analysis; this resulted in 1-bp deletion call quality, with the position deviating 10 bp from the prepared deletion being 0.8. The difference in quality score arising due to the introduction of the margin converged to 0 as the SV size became larger.

### The proposed pipeline for calling deletions

**Figure 3:**
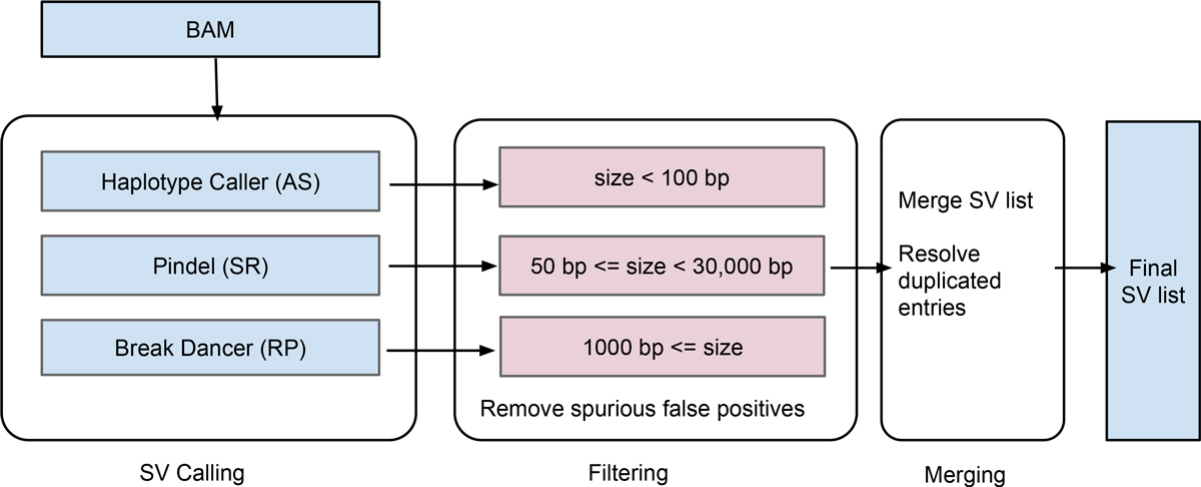
**iSVP for deletion calling**. In iSVP, each SV caller is first executed in parallel with a given BAM file, and then the results of the callers are filtered and converted in successive filtering processes. Finally, these results are merged into a unified list of deletion calls in the BED format. The parameters described in the filtering process are determined by the evaluation of simulation data. AS, SR, and RP stand for assembly, split read, and read pair approaches, respectively.

As shown in Figure 3, iSVP consists of three steps: 1) SV calling, 2) filtering, and 3) merging. In the SV calling step, we employ selected tools with different algorithms in parallel to detect a whole range of deletion sizes. Next, in the filtering step, we extract information such as the SV type, called position, and size from each caller's output. We only utilize deletion calls whose size is within a predefined range that is determined to keep precision better than 90% from simulation data analysis (the parameters employed are described in Figure 3). In the merging step, we first convert results from each caller to an extended BED format, which is convenient to compare overlaps of calls. In order to remove duplications, we remove one of the SV candidates whose precision is lower than the other if they overlapped each other by more than two thirds of their called regions. The precision for each call is determined by called size based on simulation results, and the detection of overlaps between calls is performed using BEDTools [30]. Finally, we merge the results into a unified SV call list.

## Results

### Simulation data analysis

We evaluated the performance of each tool in detecting deletions in terms of precision, recall, and their harmonic mean (F-measure) with simulation data of varying deletion sizes and read coverages. The evaluation results with each tool for read coverage 30× are summarized in Figure 1. Notably, HC predicted deletions highly accurately with precision >90% for sizes <100 bp. Since the method is an AS approach, this result suggests that the local *de novo *assembly algorithm around deleted regions was successful for relatively short deletion sizes. Pindel performed better than other methods in terms of precision for deletion sizes between 100 bp and 30,000 bp, retaining >90% precision and >90% recall. This result suggests that an SR approach, in which split reads were used for identifying breakpoints of deletions, was effective for identifying medium-size deletions. For deletion sizes >1,000 bp, BD and Delly performed comparably well, with precision >90% and recall >90%. These similar performances were possibly explained by the fact that they employ similar computational algorithms (read pair approach). The recall of Delly was better than that of BD in our analysis.

Based on the evaluation of deletion calls with each tool for simulation data, we determined the ranges of deletion size used in the filtering step of iSVP (see Figure 3). We used BD, Pindel, and HC in the SV calling step of iSVP. Although the recall of Delly was better than that of BD for simulation results (see Figure 1), we used BD because the method showed slightly better precision in longer SV regions. As we will discuss in the section on computational resources, HC needs more central processing unit (CPU) time and memory space than Pindel, and Pindel can also detect deletion sizes <100 bp with high precision and recall (see Figure 1). However, HC has even more precise calls in the region, and also determines the ploidy of each call, which is not estimated by Pindel.

**Figure 4:**
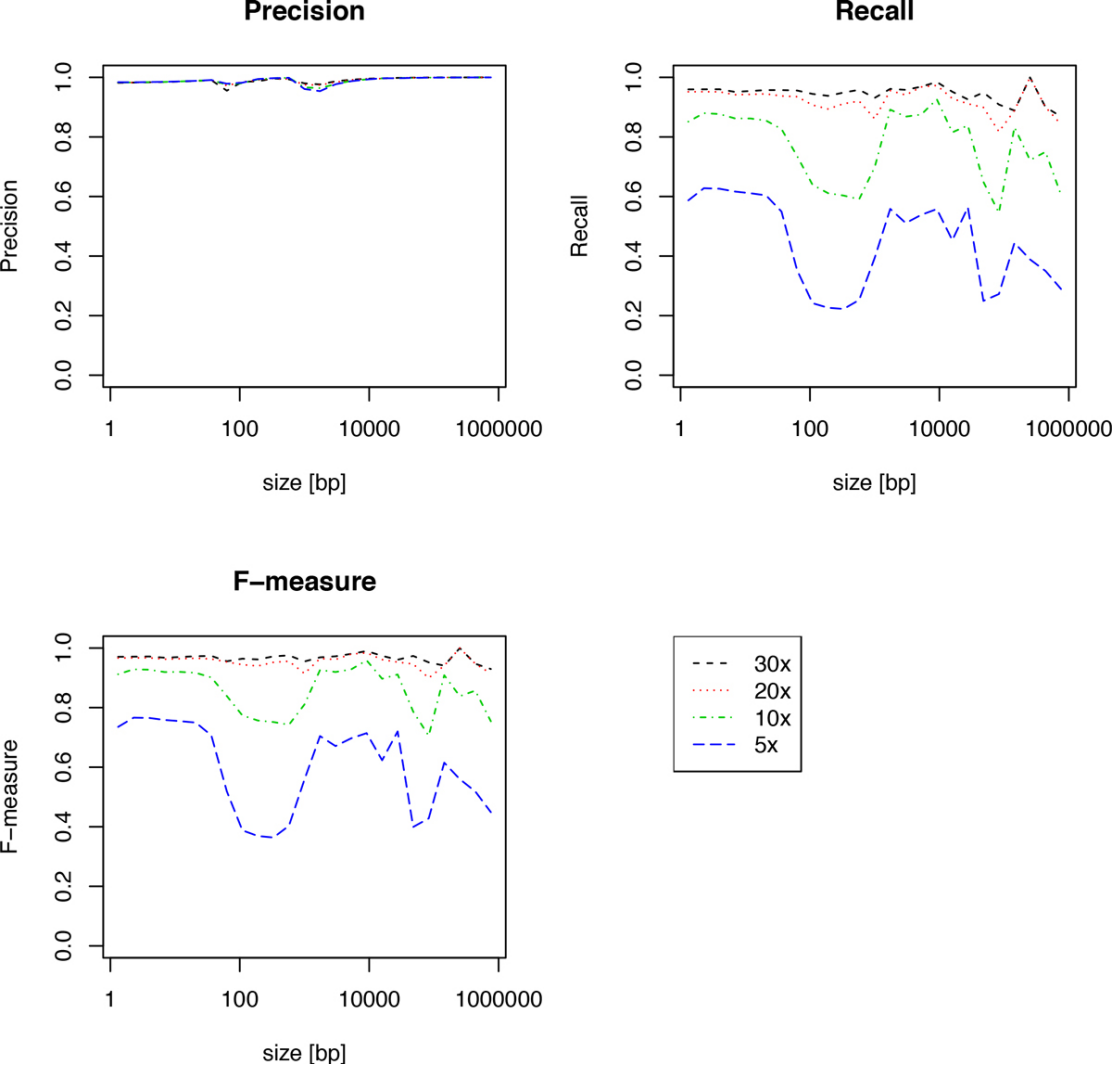
**Comparing deletion calling performance by varying depths of coverage**. The left-top, right-top, and left-bottom panels show the precision, recall, and F-measure of deletion calls, respectively, for simulation data with average depths of 5×, 10×, 20×, and 30×.

We confirmed that iSVP succeeded in achieving >90% precision and recall for almost all sizes of deletions when the average coverage of depth was 20× and 30×, as shown in Figure 4. We also found that it was hard to achieve precision and recall >90% at the same time for sequence data with average coverages lower than 10×. The result showed that the depth of coverage was consistently effective for almost all deletion sizes. Therefore, sequencing data of high coverage was essential for detecting deletions accurately and comprehensively.

### Real data analysis

**Figure 5:**
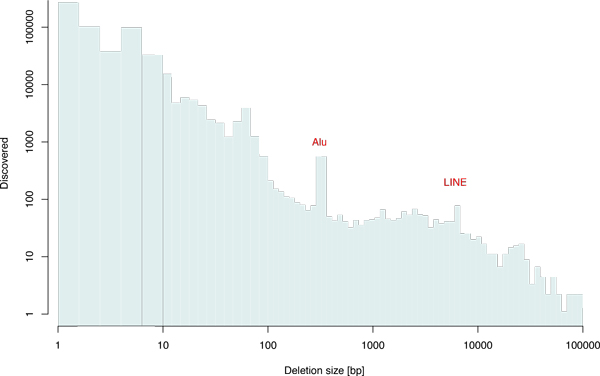
**Predicted deletions in the NA12878 sample**. A histogram of predicted deletions with iSVP using NA12878 whole-genome sequence data (45×) of 100-bp paired-end reads. The number of deletions exponentially decreased with deletion size. In addition, notable peaks around 300 bp and 6,000 bp were observed, which correspond to Alu and LINE elements, respectively.

We obtained the whole genome sequence of HapMap sample NA12878 from Illumina HiSeq 2000. The 100-bp paired-end data with an average depth of 45× was kindly provided by Illumina Inc. We applied iSVP to the NA12878 data and predicted a total of 398,518 deletions whose size ranged from 1 bp to 1,000,000 bp. The histogram of predicted deletion calls with iSVP is shown in Figure 5. It should be noted that the number of deletions exponentially declined with increasing deletion size. In addition, notable peaks around 300 bp and 6,000 bp were found, which correspond to Alus and long interspersed nuclear elements (LINEs), respectively. These peaks were also found and reported in the 1000 Genomes Project [29].

In order to evaluate the prediction results of iSVP in comparison to those of other methods, we compared these results with those of the experimentally validated deletion sets from studies by Mills [25], Conrad [23], and Kidd [24]. The number of predictions with each method that was also validated by these studies is shown in Table 2. Here, we defined true positive calls if their quality scores (see Methods section) were more than 0.9. The typical deletion sizes in the Mills, Conrad, and Kidd data were around 300 bp, 5,000 bp, and 5,000 bp, respectively. HC could not find most of the validated deletions because the AS algorithm by nature has difficulty finding relatively long deletions. iSVP and Pindel performed well with the Mills dataset, compared to BD and Delly. On the other hand, iSVP, BD, and Delly performed better than Pindel with the Conrad and Kidd datasets, as expected.

**Table 2:** Validation of deletion callings from NA12878 data.

Tool	Called (≥50 bp)	Mills (*n *= 79)	Conrad (*n *= 351)	Kidd (*n *= 58)
BD	5,014	13	158	49
Delly	286,289	13	168	51
Pindel	7,265	28	143	33
HC	1,880	4	0	0
**iSVP**	**8,130**	**30**	**166**	**49**

The sensitivity of deletion calling for each tool was confirmed by comparing to multiple datasets from the literature (Mills [25], Conrad [23], and Kidd [24]). The *n *in parentheses shows the number of total validated deletions for each experiment. BD and HC stand for BreakDancer and Haplotype Caller, respectively.

Although the numbers of true positives obtained using Delly for the validated sets were close to those obtained using BD, the number of deletion calls with sizes >50 bp was significantly larger than that seen with BD, as shown in Table 2. This indicates that excessive numbers of false positives might have been called with Delly. We examined the deletions only called by Delly and found that, they consist of calls supported by a few (2 or 3) reads, or reads of low mapping quality. As expected from the simulation data analysis, iSVP outperformed BD, Pindel, and HC for all the datasets, verifying that our approach is effective and robust for deletion calling from real data analysis.

### Computational resources

In our analysis, we used Red Hat Enterprise Linux Server release 6.2 operating system with Intel Xeon CPU E5-2670 processors running at 2.60 GHz. For each SV calling tool, the required computational resources for SV detection from the simulation data with an average depth of 30× and real data (NA12878 whole genome sequence data with average depth of 45×) are summarized in Table 1. As mentioned in the Background section, the largest amount of CPU time was required for AS, followed by SR and RP, using simulation data. By comparing the results of simulation and real data, we see that BD and Pindel required predictable amounts of CPU time and memory space based on the simulation data (i.e., nearly proportional to the coverage of read depth). For HC, we found that the CPU time was several times larger than expected. Delly required relatively larger resources in terms of CPU time and maximum memory size for the real data (see Table 1). For iSVP, most of the computational resources that iSVP use are in the SV calling step. The CPU time and memory space consumed for the successive filtering and merging steps are less than 30 minutes and 2 Gb, respectively.

## Discussion and conclusions

We investigated several types of SV calling tools and evaluated their performance with a detailed simulation analysis. We found that there were significant differences in performance according to the employed algorithms and deletion size. Each tool had its strength and weakness, and there was no algorithm that consistently outperformed others. HC, an AS approach, performed especially well for deletions in the size range 1-100 bp. Pindel, an SR approach, performed relatively better than other methods for deletions of 100-10,000 bp. BD and Delly, both RP aproaches, were able to detect large deletions. Importantly, regardless of the algorithm used, high-coverage reads were consistently informative for detecting deletions. Based on the simulation results, we developed iSVP, a new pipeline to unify these methods with filtering and merging processes to comprehensively and reliably detect genomic SVs. Our approach succeeded in achieving more than 90% precision and 90% recall for a broad range of deletion sizes. We showed that a relatively higher depth of coverage (more than 20×) was required to gain good performance in SV detection from simulation experiments. This high-coverage requirement may be one of the reasons why comprehensive catalogs of SVs are still limited at the moment.

By applying iSVP to human whole genome sequence data from a HapMap NA12878 sample, we detected numerous SVs that were biologically explainable, and some of them have been validated by other independent experiments. iSVP is broadly applicable to high-coverage whole genome sequencing data with reasonable computational resources, which will enhance the genome-wide detection of SVs for the identification of disease-causing variants. However, the number of recalls from real data was smaller than that expected from the computational simulation. This problem may be related to the complexity of sequences around the SVs, which has not been sufficiently investigated yet.

Our future work will include a study of the performance of iSVP for other various types of SVs other than deletions, such as insertions, duplications, and translocations, which are more difficult to detect and validate. Furthermore, developing a pipeline to genotype multiple samples simultaneously is also a challenging and promising task.

## Competing interests

The authors declare that they have no competing interests.

## Authors' contributions

TM, NN, and MN conceived the study, TM, NN and KK designed the computational experiment, TM performed the analysis, and TM, NN, KK, and MN interpreted the results. MT, AO, YS, and YYK collaborated on data collection and interpretation of the results. TM, NN, KK, YS, YYK, and MN wrote the manuscript. All the authors read and approved the final manuscript.

## Declarations

The publication costs for this article were funded by the MEXT Tohoku Medical Megabank Project.

This article has been published as part of *BMC Systems Biology *Volume 7 Supplement 6, 2013: Selected articles from the 24th International Conference on Genome Informatics (GIW2013). The full contents of the supplement are available online at http://www.biomedcentral.com/bmcsystbiol/supplements/7/S6.
